# An interaction‐driven cannibalistic reaction norm

**DOI:** 10.1002/ece3.3801

**Published:** 2018-01-27

**Authors:** Kinya Nishimura

**Affiliations:** ^1^ Graduate School of Fisheries Sciences Hokkaido University Japan

**Keywords:** cannibalistic dimorphism, density‐driven expression, exploitative competition, geometric morphometrics, *Hynobius retardatus*, interaction‐driven expression, interference competition, reaction norm

## Abstract

Cannibalism is induced in larval‐stage populations of the Hokkaido salamander, *Hynobius retardatus*, under the control of a cannibalism reaction norm. Here, I examined phenotypic expression under the cannibalism reaction norm, and how the induction of a cannibalistic morph under the norm leads to populational morphological diversification. I conducted a set of experiments in which density was manipulated to be either low or high. In the high‐density treatment, the populations become dimorphic with some individuals developing into the cannibal morph type. I performed an exploratory analysis based on geometric morphometrics and showed that shape characteristics differed between not only cannibal and noncannibal morph types in the high‐density treatment but also between those morph types and the solitary morph type in the low‐density treatment. Size and shape of cannibal and noncannibal individuals were found to be located at either end of a continuum of expression following a unique size–shape integration rule that was different from the rule governing the size and shape variations of the solitary morph type. This result implies that the high‐density‐driven inducible morphology of an individual is governed by a common integration rule during the development of dimorphism under the control of the cannibalism reaction norm. Phenotypic expression under the cannibalism reaction norm is driven not only by population density but also by social interactions among the members of a population: variation in the populational expression of dimorphism is associated with contingent social interaction events among population members. The induced cannibalistic morph thus reflects not only by contest‐type exploitative competition but also interference competition.

## 1 INTRODUCTION

An organism's phenotype is the end product of a complex series of developmental processes. Organisms can develop alternative phenotypes depending on many contingent events occurring during their lifetime (Gilbert & Epel, 2009; Piersma & van Gils, 2010). The reaction norm, which refers to the set of phenotypes that can be expressed by a single genotype in individuals exposed to different environmental conditions, is a useful concept for understanding adaptive phenotypic plasticity (Sarkar, 2001; Schlichting & Pigliucci, 1998; West‐Eberhard, 2003).

Social status‐driven sex determination (Frike & Frike, 1977; Godwin, Luckenbach, & Borski, 2003; Warner & Swearer, 1991), caste divisions (Wheerer, 1991), and alternative life histories supporting different mating strategies (Gross, 1991) are population‐level expressions of a reaction norm involving a phenotypic plasticity in which individuals develop into one or another alternative phenotype, for example, into a male or a female, in response to intraspecific interactions. The expression of alternative phenotypes by members of a population has intriguing evolutionary implications, and various attempts have been made to describe the evolutionary conditions that lead to the development and coexistence of alternative phenotypes in a population (Brockmann & Taborsky, 2008; Clutton‐Brock, Albon, & Guinness, 1986; Giraldeau & Livoreil, 1998; Gross, 1996; Maynard Smith, 1982; Pfennig & Pfennig, 2012).

Cannibalism is a facultative resource acquisition strategy that is induced in response to various external environmental stimuli and internal conditions (Fox, 1975; Polis, 1981). In some species, cannibalism is associated with dimorphic populations, in which some members develop an aggressive exploitative dominant cannibal‐type and the other members develop a less aggressive subordinate noncannibal‐type (Bragg, 1956; Folkvord, 1997; Hardie & Hutchings, 2014; Orton, 1954; Powers, 1907). Much attention has been focused on the characteristics of induced cannibals (Collins & Cheek, 1983; Lannoo & Bachmann, 1984; Pierce, Mitton, Jacobson, & Rose, 1983; Walls, Belanger, & Blaustein, 1993), proximate and social factors facilitating or restraining the induction of cannibals (Collins & Cheek, 1983; Heermann, Scharf, van der Velde, & Borcherding, 2013; Hoffman & Pfennig, 1999; Pfennig & Collins, 1993), and the induction rate of cannibals within and among populations (Michimae & Wakahara, 2002). Thus, cannibalism has been studied in the context of a reaction norm for the inducibility of cannibalistic phenotypes (Fox, 1975; Michimae, 2006; Polis, 1981; Tayeh et al., 2014).

However, these studies focused only on the inducibility of the cannibalistic phenotype; they did not investigate the implications of the reaction norm holistically. Interactions among the members of a group do not only induce the cannibalistic phenotype in some group members; they also determine the phenotypes of other members of the group in such a way that the group becomes dimorphic. Thus, to gain a holistic understanding of the reaction norm, we must be aware that the phenotypes of all interacting group members are governed by the reaction norm and examine populational phenotypic patterns.

Even if all individuals share identical or, at most, subtly different states with regard to body size and physiological condition at the start of development, under the cannibalism reaction norm, behavioral and morphological outcomes will differ among group members. Contingent social interaction events constitute complex causative factors initiating heterogeneous expression of phenotypes under the reaction norm among individuals, causing them to begin to deviate from the initial identical state, and subsequent interactions then become asymmetrical among the members. Contingent interaction events among the members with different states drive phenotypic expression under the reaction norm; thus, they determine the final phenotype of each individual and cause the population to become dimorphic. Therefore, once the cannibalistic phenotype is expressed in a population, it should function to promote differentiation of group members into cannibal and noncannibal types, particularly with respect to individual size and possibly shape. Phenotypic expression under the cannibalism reaction norm can be regarded as being primarily driven by interactions among population members, and these interactions give rise to intracohort cannibalism and dimorphism in a population. In this paper, I consider the cannibalism reaction norm in the context of the populational morphological expression of phenotypic plasticity.

In several amphibian species, cannibalism occurs at the larval stage (Crump, 1992) and is accompanied by the differentiation of group members into cannibalistic and noncannibalistic phenotypes (Bragg, 1956; Crump, 1992; Newman, 1989; Powers, 1907; Walls, Beatty, Tissot, Hokit, & Blaustein, 1993). The well‐established cannibalistic phenotype is suited for macrophagous feeding because it characteristically has a large head and a wide, enlarged jaw (Lannoo & Bachmann, 1984; Ohdachi, 1994; Pierce et al., 1983; Powers, 1907). The major explanatory factor for the phenotypic differentiation of the group members is not genotypic polymorphism (Pierce, Mitton, & Rose, 1981) but plastically expressed phenotypic polymorphism (Collins & Cheek, 1983; Lannoo & Bachmann, 1984; Orton, 1954; Pfennig, 1990; Wakahara, 1995). Therefore, amphibian larvae are appropriate materials for investigating how the cannibalism reaction norm gives rise to cannibalism and a dimorphic population consisting of cannibalistic and noncannibalistic phenotypes.

The Hokkaido salamander, *Hynobius retardatus*, is a well‐studied model system of cannibalistic dimorphism under the control of a reaction norm (Nishihara, 1996a,b; Wakahara, 1995). Under high‐density conditions, cannibalism frequently occurs and dimorphism becomes established as larval development progresses (Wakahara, 1995). A well‐developed cannibalistic dimorphic population consists of large, robust cannibalistic larvae and smaller, more slender larvae (Wakahara, 1995).

In this study, I examined population‐level morphological expression associated with cannibalism in larvae of *H. retardatus* by conducting a set of experiments in which the density of *H. retardatus* larvae was manipulated. I first investigated developing morphological features of the larvae associated with the experimental high‐ and low‐density manipulations. Next, I investigated the contingent social events that were associated with the morphological diversification of the larval populations and the correlation of these events with populational morphological distributions. I expected cannibalism to emerge under the high‐density manipulation and that the larvae would develop either a cannibal or a noncannibal morph type in response to contingent social interaction events in the population. I used geometric morphometrics to analyze the emerging morphological characteristics and to clarify phenotypic expression under the cannibalism induction reaction norm. I elucidated how the cannibalism induction reaction norm manifests in individuals and how it generates consequently a populational morphological structure characterized in the cannibalistic dimorphism.

## 2 MATERIALS AND METHODS

I collected fertilized egg clutches of the salamander *H*.* retardatus* from ponds in the vicinity of Hakodate (N 41°53′, E 140°34′), Hokkaido, Japan, in early April 2014. Every 10 or so egg clutches were placed together in stock tanks filled with dechlorinated tap water. Water temperature in both the stock and experimental tanks was held at 16°C, and the tanks were kept in a laboratory on a natural light/dark schedule.

### 2.1 Induction experiment

I set two rearing conditions, a high‐density condition and a solitary condition, so that the occurrence of cannibalism and the induced morph types would differ between them. The solitary treatment was used as an extreme low‐density condition to assure the nonoccurrence of the cannibal morph type.

For the high‐density treatment, I prepared 10 experimental tanks (base area, 23 cm × 35 cm; depth, 14 cm), each with about 4 L of dechlorinated tap water. I then picked out from the stock tanks simultaneously hatched larvae from at least 30 different clutches and randomly allocated 30 individuals to each of the 10 experimental tanks.

For the solitary treatment, I prepared 30 experimental tanks (base area, 15 cm × 28 cm; depth, 8.5 cm), each with about 1.5 L of dechlorinated tap water, and allocated one larva to each of the tanks.

I treated the group of individuals in each tank of the high‐density treatment as a “population,” and I treated all individuals in the solitary treatment as members of a single hypothetical extremely low‐density “population,” in which individuals are assumed to never encounter one other. Thus, I ideationally used the word “population” for groups of individuals.

The total body length of a subsample of hatchlings was 15.88 ± 0.793 mm (mean ± *SD*,* n *=* *20). Every 3 days, the larvae were fed a sufficient number of live freshwater oligochaetes (Tubifex), and any food remaining in their tanks after 24 hr was removed. The water in the tanks was replaced every 3 days with fresh water.

I ended the induction experiment after 12 days. In each high‐density treatment tank, I counted the number of missing larvae, which I considered to be the number of cannibalized victims. In both experimental treatments, I photographed every surviving individual in dorsal and lateral view with two digital cameras. Most of the following analyses were conducted using the digitized data of the photographs after calibration for scale in millimeters to two decimal places.

### 2.2 Determination of cannibals and noncannibals

Cannibalism occurred in all high‐density treatment tanks. Generally, a cannibal swallows the whole body of a live victim from the head. In most cases, I did not observe the actual moment of attack of one individual on another. Therefore, at unknown times in the past of the development, one perpetrator of cannibalism may have eaten more than one victim, whereas another may have eaten only one victim. This disparity in the timings and the number of victims eaten can result in some cannibals developing a marked cannibal morph type and other cannibals not completing the development of the cannibal morph type.

Therefore, it was necessary to deal with the possibility that identification of cannibals in each tank would not be straightforward. Initially, I used two criteria to visually identify a number of individuals as cannibals. First, I used circumstantial evidence to identify cannibals. Any individual whose stomach contained a conspecific, which was possible to confirm visually from the outside, without dissecting or opening up the body, was obviously a cannibal. Then, in addition, I identified individuals with an enlarged, well‐developed jaw and large body as cannibals. It is reasonable to identify such individuals as cannibals not only because large‐jawed individuals are well suited to a cannibalistic feeding habit but also because such a large body can only be acquired in a short time by a larva that has preyed on large, nutritious conspecific prey (Nishihara, 1996a; Wakahara, 1995).

However, some cannibals were clearly missed by these criteria, because in some tanks in which some individuals had in fact been cannibalized, no cannibals could be visually identified. Given the inadequacy of this first screening result, another means of distinguishing cannibal individuals from noncannibal individuals was required. Although some studies have used a simple length ratio criterion such as a jaw width/head width ratio >0.9 (Michimae, 2006; Michimae & Wakahara, 2002), I established a criterion based on length measurements of multiple body dimensions that were used as input to a machine‐learning classification algorithm; then, I screened the larvae for additional cannibals with the aid of this classification algorithm (Appendix).

### 2.3 Morphological analyses

**Figure 1:**
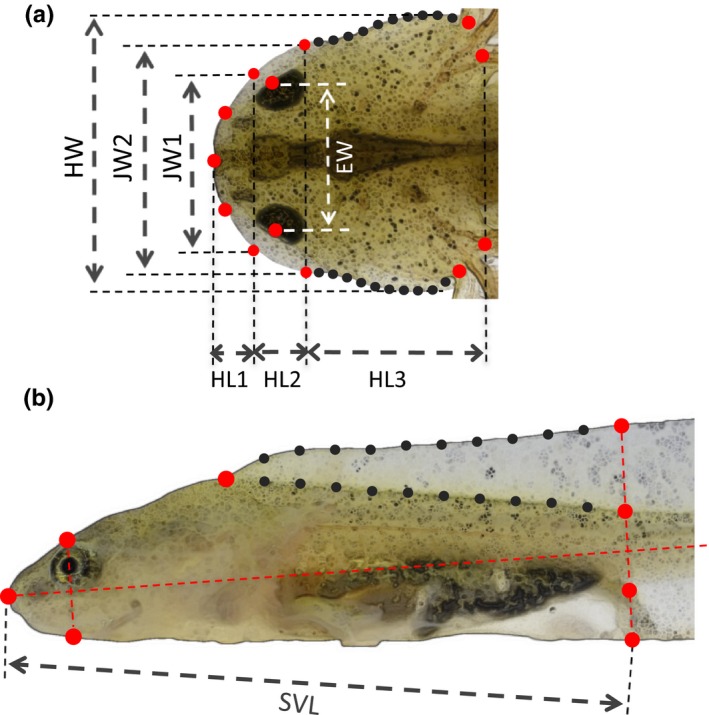
Landmarks (red dots) and semilandmarks (black dots) for the geometric morphometric analysis. Semilandmarks were spaced at equal intervals between landmarks. (a) Dorsal head profile showing the seven body dimensions measured: HW, JW1, JW2, EW, HL1, HL2, and HL3. (b) Lateral body profile. The snout–vent length, SVL, was measured

To rigorously quantify shape diversity, I conducted a landmark‐based geometric morphometric analysis (Bookstein, 1991; Rohlf & Marcus, 1993; Zelditch, Swiderski, & Sheets, 2012). Landmarks are specific points on a biological form established according to rules commonly accepted by the morphometrics community (Bookstein, 1991; Zelditch et al., 2012). I placed landmarks to describe fundamental body shape characteristics on dorsal and lateral views of the larval body (Figure 1). The caudal fin was excluded from the lateral profile because in the high‐density treatment, it was frequently damaged or partially lost by being bitten off by other individuals.

Because important features of morphological variations may not be sufficiently captured using only landmarks, I also placed semilandmarks to describe the curvature of some parts of the body profile. Landmarks and semilandmarks were placed on the photographic images using tpsDig software (Rohlf, 2005).

The sets of landmarks that I used included different types, as defined by Bookstein (1991). The theoretical validity of various methods for analyzing data composed of different types of landmarks and semilandmarks has been examined (Bookstein, 1997; Zelditch et al., 2012), but all proposed methods are controversial (MacLeod, 2013; Zelditch et al., 2012). Following the recommendation of MacLeod (MacLeod, 2013), I lumped all types of landmarks and semilandmarks together without distinction for analytical practicality.

I prepared the data using a standard landmark‐based geometric morphometrics procedure (Dryden & Mardia, 1998). On the dorsal profile of the head, bilateral pairs of landmarks and semilandmarks were symmetrized with respect to the body axis by averaging their coordinates, and the landmark configurations were subjected to a Procrustes superimposition before the shape analysis to remove the effects of size and position (Zelditch et al., 2012). Just one side of each configuration was used in the shape analysis, although both sides of a symmetric configuration are depicted in the figures. Furthermore, the symmetric data of both sides were used to calculate the head centroid size (HeadCS), a one‐dimensional parameter defined as the square root of the sum of the squared distances from each landmark and semilandmark to the centroid of the head landmark and semilandmark coordinates (Dryden & Mardia, 1998). The configurations on the lateral profile of the body were also subjected to a Procrustes superimposition to remove the effects of size, position, and orientation before the morphometric analyses.

The Procrustes superimpositions and the subsequent morphological analyses were conducted using the MorphoJ software (Klingenberg, 2011), Geometric morphometrics for Mathematica (Polly, 2014), and custom‐made programs for Mathematica.

## 3 RESULTS

### 3.1 Determination of cannibals and noncannibals

In the experimental tanks of the high‐density treatment, at least one and a maximum of nine individuals per tank (4.7 ± 2.65 ind/tank, mean ± *SD*) had been eaten by one or more of the survivors in each tank. By the visual criteria and screening with the aid of a machine learning classification algorithm (Appendix Table S1), I identified 25 individuals as cannibals and 221 individuals as noncannibals among the surviving individuals in the high‐density treatment (Appendix Fig. S1). The experimental tanks of the high‐density treatment each held from one to six cannibal individuals (2.5 ± 1.509 ind/tank, mean ± *SD*), and at least one and a maximum of nine individuals per tank (4.7 ± 2.65 ind/tank, mean ± *SD*) had been cannibalized.

Henceforth, I use the category labels Cannibal and NonCannibal for cannibal and noncannibal individuals in the high‐density treatment, and I use the category label Solitary for individuals in the solitary treatment.

### 3.2 Size and shape among the categories

#### 3.2.1 Shape

To delineate shape variation among all larvae, I conducted an integrative shape analysis using the Procrustes coordinates data of the dorsal head and lateral body profiles.

Various indices have been introduced for quantification of the degree of morphological integration (Armbruster, Pélabon, Bolstad, & Hansen, 2014; Bookstein & Mitteroecker, 2014; Garcia, 2012; Smilde, Kiers, Bijlsma, Rubingh, & van Erk, 2009). I explored shape integration using Escoufier's coefficient (Zelditch et al., 2012) and a block correlation method based on partial least squares (PLS) (Rohlf & Corti, 2000).

Even though the lateral body profile included the larva's head, the dorsal head and lateral body profiles did not share any intersecting landmark configurations, and the projection planes of the landmarks were mutually orthogonal. Therefore, dorsal profile landmarks and lateral profile landmarks were geometrically mutually independent and provided different information to the overall body shape analysis.

I calculated Escoufier's coefficient, RV, between the head shape Procrustes coordinates and the body shape Procrustes coordinates, and obtained a statistically significant nonzero value: RV = 0.2439, *p *<* *.0001 (a within‐block permutation test (*n *=* *10,000) of the null hypothesis that RV = 0). This result implies an overall correlative variation between the dorsal projection of head shape and the lateral projection of body shape in the pooled data of the three categories.

I also applied a PLS analysis to the Procrustes coordinates on the dorsal head shape projection and the lateral body shape projection. The first PLS axes accounted for 85.71% of the total squared covariance between the dorsal and lateral shape blocks. The PLS1_DorsalShape_ scores and PLS1_LateralShape_ scores were correlated as follows: *r *=* *0.672 (a permutation test (*n *=* *10,000) of the null hypothesis that *r *=* *0, *p *<* *.0001).

**Figure 2:**
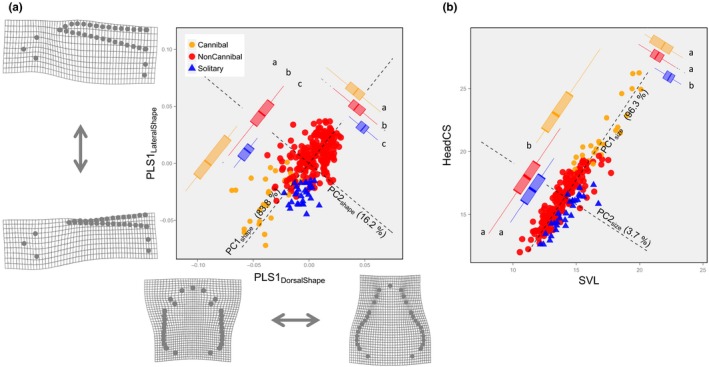
(a) Integration of lateral and dorsal shape scores by partial least squares (PLS) analysis. The joint distribution of PLS1 scores of the dorsal head shape projection and of lateral boy shape projection is shown on the PLS1 plane. The deformation profiles on the grids demonstrate how the shapes change along each PLS1 axis. (b) Integration of SVL and the HeadCS. The joint distribution of SVL and HeadCS is shown. The dominant (PC1) and secondary (PC2) principal component axes are shown by dashed lines: in (a), PC1_Shape_ and PC2_Shape_ and in (b), PC1_Size_ and PC2_Size_. The box plots summarize the projected PC scores. For more details, see the text

I examined the shape changes along the PLS1 axes by plotting the deformation profiles on grids along each PLS axis (Figure 2a). From the deformation profiles, it can be seen that there is shape change continuum along the PLS1_DorsalShape_ axis between a tetragonal and a trigonal head shape, and another along the PLS1_LateralShape_ axis between a deep and a shallow dorsal fin. The correlation between the dorsal head shape projection and the lateral body shape projection implies that an integration rule for head shape and body shape (particularly dorsal fin shape) variation exists. This integration rule is summarized in a single dimension by the major axis regression line (i.e., the dominant axis of a principal component analysis, PC1_Shape_), where PC1_Shape_ accounts for 83.8% of the total variance in the distribution on the PLS1_LateralShape_–PLS1_DorsalShape_ plane. The PC1_Shape_ score variation indicates that a tetragonal head shape is related to a shallow dorsal fin, and a trigonal shape head is related to a deeper dorsal fin (see Figure 2a).

Cannibal individuals had lower scores on the PC1_Shape_ axis, reflecting their tetragonal shape head (i.e., the anterior part of head was widened by the development of a large jaw) and shallow dorsal fin. NonCannibal individuals had higher scores on the PC1_Shape_ axis, reflecting their trigonal shape head and deep dorsal fin. Solitary individuals had intermediate scores and were distributed in between the other two groups on the PC1_Shape_ axis. In sum, a small PC1_Shape_ score implies a cannibal‐like shape, and a large PC1_Shape_ score implies a NonCannibal‐like shape.

The PC2_Shape_ axis, which is orthogonal to PC1_Shape_, accounted for only 16.2% of the total variance, but the among‐group variation was systematic along the axis. PC2_Shape_ scores of Cannibal, NonCannibal, and Solitary individuals were distributed in descending order along the axis; this result indicates that, with the major shape effect captured by PC1_Shape_ axis removed, head shape tends to be tetragonal and the dorsal fin tends to be deep in that order (see Figure 2a).

#### 3.2.2 Size

I explored size variations using two independent indices: snout‐vent length (SVL, an index of body length) and head centroid size (HeadCS, a one‐dimensional index of head size). As would generally be expected, SVL and HeadCS variations were highly correlated (*r *=* *0.9149, *t *=* *37.37, *df* = 272, *p *<* *.000). The correlative variations could be summarized in a single dimension by the major axis regression line (PC1_Size_), where PC1_size_ accounts for 96.3% of the total variance of the distribution on the SVL–HeadCS plane (Figure 2b). PC1_size_ primarily indicates overall body size relationship with a long body relating to a large head, and a short body to a small head.

The PC1_Size_ scores suggest that Cannibal individuals had larger heads and longer bodies than NonCannibal and Solitary individuals. The minor axis, PC2_Size_, accounting for 3.7% of the total variation, indicated a systematic variation among groups. After removal of the major size effect captured by PC1_Size_, Solitary individuals had a longer body and a smaller head compared with both Cannibal and NonCannibal individuals (see Figure 2b).

#### 3.2.3 Size–shape

**Figure 3:**
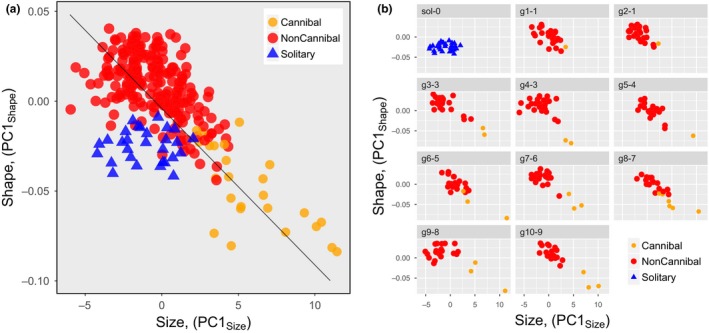
(a) Size–shape distributions of Cannibal, NonCannibal, and Solitary individuals. Size (PC1_Size_) and shape (PC1_Shape_) scores are the same as the PC1 axis scores in Fig. 2. (b) The overall size–shape distribution subdivided into the distributions in each tank population. The label at the top of each grid indicates the tank population ID (g1, g2, etc.) and, following the hyphen, the numbers of cannibalism victims in that population. “sol‐0” represents the hypothetical population with no victims, consisting of all individuals in the solitary treatment

Finally, I examined the interactive pattern of the size and shape variations. The size–shape relation can be summarized by comparing the PC1_Size_ and PC1_Shape_ scores of the three categories (Figure 3a). Size and shape variations in the pooled data of the three categories varied correlatively (*r = *.6486, *t *=* *14.053, *df* = 272, *p *<* *.0000). I conducted identity tests of the slope and intercept parameters of the estimated regression lines of the larvae in the high‐density treatment categories, Cannibal and NonCannibal, by a standardized major axis regression (Warton, Duursma, Falster, & Taskinen, 2012). The results showed that the Cannibal and NonCannibal regression lines not only had the same slope (LL = 0.14, *df* = 1, *p *<* *.6986) but also had the same intercept (Wald statistics = 0.6041, *df* = 1, *p *<* *.4370), implying that a unique integration rule explains the size and shape variations of the members in the cannibalism (high‐density) populations. This morphological diversification can be interpreted as the density‐driven phenotypic expression pattern under the cannibalism reaction norm of this species. In contrast, the larvae in the Solitary category exhibited no size–shape correlation (*r *=* *0.03406, *t *=* *0.1771, *df* = 27, *p *<* *.8609).

### 3.3 Shape discrimination among the categories

**Figure 4:**
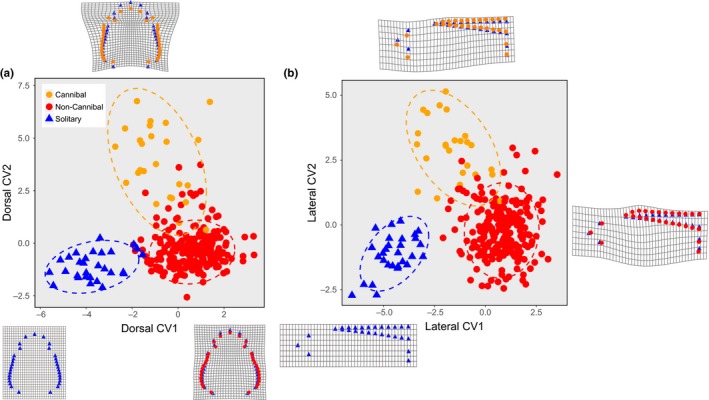
Canonical variable (CV) scores for (a) dorsal head shape and (b) lateral body shape. Deviations of the mean NonCannibal shape and the mean Cannibal shape from the mean Solitary shape are shown on the grids

The alignments of the shape and size distributions of the Cannibal, NonCannibal, and Solitary categories (Figures 2 and 3a) imply that the three categories are morphologically discriminable entities. To quantify differences in mean shape among the three categories, I conducted pairwise permutation tests of the Procrustes distance for pairs of categories, separately for the dorsal and lateral profiles. Individuals in Cannibal, NonCannibal, and Solitary categories had distinct shapes in both dorsal and lateral profiles (Table 1). Figure 4 showed the discriminability of the dorsal head shape and the lateral body shape of the three categories on canonical variate analysis planes.

**Table 1:** Procrustes distances between mean shapes and permutation test results

	Procrustes distances between category means			*p*‐values from permutation tests (10,000 permutation rounds)	
	Cannibal	NonCannibal		Cannibal	NonCannibal
Dorsal shape					
NonCannibal	0.0739	–	NonCannibal	*p *<* *.0001	–
Solitary	0.0860	0.0258	Solitary	*p *<* *.0001	*p *<* *.0001
Lateral shape					
NonCannibal	0.0429	–	NonCannibal	*p *<* *.0001	–
Solitary	0.0311	0.0390	Solitary	*p *<* *.0001	*p *<* *.0001

### 3.4 Interaction‐driven populational expression of the cannibalism reaction norm

#### 3.4.1 Morphological distributions

In the solitary treatment, individuals were subjected to neither mutual interactions nor actual predation; thus, the experiences of the individuals were rather uniform. In contrast, in the high‐density treatment, there were from one to six Cannibal individuals and from 21 to 29 total survivors per population of each experimental tank. Thus, in the high‐density treatment, it is reasonable to infer that the surviving individuals experienced mutual interactions, such as attacking or being attacked and the actual consumption of conspecific individuals, differently among the populations. It might be expected, therefore, that the cannibalism reaction norm would not be expressed uniformly among the populations of the high‐density treatment. I predicted that populational morphological diversification and morph‐type differentiation would be more advanced in populations with more numerous and intensive mutual interactions among members.

I examined populational morphological diversification and morph‐type differentiation among populations in relation to population processes, that is, mutual interactions between individuals and contingent events. Unfortunately, owing to the census scheme, past processes experienced by the individuals in each population are unknowable. However, I hypothesized that the census records of the numbers of Cannibal individuals and of cannibalized victims would reflect the intensities of the past cannibalistic mutual interactions that the surviving individuals in the population experienced during the experimental period.

First, I inferred that the cannibalism reaction norm would be expressed at the individual‐level such that, by and large, Cannibal individuals in tanks with more victims would exhibit a more Cannibal‐like shape and size (i.e., more tetragonal heads, shallower dorsal fins, and larger overall size) and NonCannibal individuals in tanks with more Cannibal individuals would exhibit a more NonCannibal‐like shape and size (i.e., more trigonal heads, deep dorsal fins, and perhaps smaller overall size).

At the population‐level, I deduced that the more Cannibal individuals or more victims that there were in a tank, the more advanced the morph‐type differentiation would be. I expected that as morph‐type differentiation progressed, the joint size and shape distribution, denoted by the PC1_Size_ and PC1_Shape_ scores (Figure 3a), would become more correlative. Furthermore, I expected marginal distributions of size or shape, or both, to become more skewed, owing to the separation of the morphological characteristics of Cannibal individuals, which would constitute a minority of the population, from the morphological characteristics of the majority NonCannibal members of the population.

Therefore, I analyzed the association between the census records (numbers of Cannibal individuals and cannibalized victims) and populational morphological characteristics among populations. Because the solitary treatment was used as an extremely low‐density condition, in this analysis, I treated Solitary individuals as composing a single hypothetical population in which individuals neither had cannibalistic interactions nor did they experience any other direct or indirect effects from other population members. This hypothetical population served as a reference for the populations of the high‐density treatments, which could have had cannibalistic interactions as well as other direct and indirect effects from the population members.

In each population, the census numbers of Cannibal individuals (*x*_1_) and their victims (*x*_2_) are the elements of vector **X **= (*x*_1_, *x*_2_), and the product‐moment correlation coefficient of the joint size–shape distribution (*y*_1_ = *r*(PC1_Size_, PC1_Shape_)), the moment coefficient of the skewness of the size distribution (*y*_2_ = *sk*(PC1_Size_)), and the moment coefficient of the skewness of the shape distribution (*y*_3_ = *sk*(PC1_Shape_)) are the elements of the distribution statistics vector **Y **= (*y*_1_, *y*_2_, *y*_3_) (Table 2).

**Table 2:** Correlation analyses between census numbers of Cannibals and victims, and the size and shape distributions statistics

	**X** = (*x*_1_, *x*_2_)		**Y** = (*y*_1_, *y*_2_, *y*_3_)		
	*x*_1_ = Cannibal	*x*_2_ = Victim	*y*_1_ = *r*(PC1_Size_, PC1_shape_),	*y*_2_ = *sk*(PC1_size_),	*y*_3_ = *sk*(PC1_shape_)
sol	0	0	0.03406	−0.1553	−0.09257
g1	1	1	−0.5806	−0.4746	−0.2221
g2	1	1	−0.5857	0.6918	−0.4554
g3	2	3	−0.8611	1.4240	−1.3146
g4	2	3	−0.5991	0.3082	−1.7123
g5	1	4	−0.8153	1.7225	−0.9942
g6	3	5	−0.8441	2.1265	−1.1261
g7	3	6	−0.8320	1.1593	−1.3087
g8	6	7	−0.8861	1.1094	−0.6786
g9	3	8	−0.6765	1.5385	−1.6399
g10	3	9	−0.9165	1.3551	−1.1827
	**X** vs. **Y** corr.		RV(**X**,** Y**) = 0.4847*, *r*_PLS1_(**X**,** Y**) = 0.7083*		
	*x*_1_ vs. **Y** corr.		RV(*x*_1_, **Y**) = 0.2481^ns^, *r*_PLS1_(*x*_1_, **Y**) = 0.5097^ns^		
	*x*_2_ vs. **Y** corr.		RV(*x*_2_, **Y**) = 0.5060*, *r*_PLS1_(*x*_2_, **Y**) = 0.7213*		
	Separate correlations		*r*(*x*_2_, *y*_1_) = −0.6845*, *r*(*x*_2_, *y*_2_) = 0.6255*, *r*(*x*_2_, *y*_3_) = −0.6035*		

*x*_1_ and *x*_2_ are the census numbers of Cannibals and victims, respectively, in each population. *y*_1_ is the product‐moment correlation of the joint PC1_size_–PC1_shape_ distributions (shown in Fig. 3b), *y*_2_ is the moment coefficient of skewness of the PC1_size_ distribution, and *y*_3_ is the moment coefficient of skewness of the PC1_shape_ distribution in each population. The significance of Escoufier's coefficient (RV), *r*_PLS1_, and *r* were evaluated by permutation tests (*n *=* *10,000) of the null hypothesis of a zero value. sol, pooled individuals of the solitary treatment; *g*, tanks of the high‐density treatment. *p <* *.0001.

First, I addressed the question of whether the census number of Cannibal individuals or victims was related in aggregate to the size–shape distribution statistics of the populations. A tangible analytical means of addressing this question is to perform a correlation analysis of the variable vectors **X** and **Y**. I again adopted two multivariate extensions of the ordinary univariate correlation method, Escoufier's coefficient and the correlation of the first PLS axes (denoted as RV(**X**,** Y**) and *r*_PLS1_(**X**,** Y**), respectively) to evaluate the correlative relationship of **X** and **Y**. Both RV(**X**,** Y**) and *r*_PLS1_(**X**,** Y**) were significantly different from zero, suggesting that the census numbers of either the Cannibals or the victims or both were related in aggregate to the correlation of the joint size–shape distribution and the skewnesses of the size and shape distributions (see Table 2).

The elements of vector **X**, that is, the census numbers of Cannibal individuals (*x*_1_) and of victims (*x*_2_), had a mutually positive correlation (*r*(*x*_1_, *x*_2_)* *= 0.7652, *t *=* *3.5657, *df* = 9, *p *<* *.006065). Then, I addressed the question of whether *x*_1_ or *x*_2_ or both were separately correlated with **Y**. RV(*x*_1_, **Y**) and *r*_PLS1_(*x*_1_, **Y**) were not significantly different from zero (see Table 2), suggesting that the census numbers of Cannibal individuals did not significantly affect the size–shape distribution patterns of the populations. In contrast, RV(*x*_2_, **Y**) and *r*_PLS1_(*x*_2_, **Y**) were significantly different from zero (see Table 2), suggesting that the census numbers of victims had a significant effect on the size–shape distribution patterns of the populations.

Next, I examined the correlation between *x*_2_ and each element of **Y**, separately. The correlations between *x*_2_ and each separate element of **Y** were significantly different from zero (see Table 2). In view of the inferred meanings of PC1_size_ and PC1_Shape_, the negative value of *r*(*x*_2_, *y*_1_) indicates that as the census number of victims became larger, the correlation of the joint size–shape distribution became higher such that smaller size tended to be more closely associated with a trigonal head and a deep dorsal fin and larger size tended to be more closely associated with a tetragonal head and a shallow dorsal fin. (see Figure 3b). The positive value of *r*(*x*_2_, *y*_2_) indicates that as the census number of victims became larger, the skewness of the size distribution became larger such that the size of Cannibal individuals became proportionately much larger than the size of the NonCannibal individuals. The negative value of *r*(*x*_2_, *y*_3_) indicates that as the census number of victims became larger, the skewness of the shape distribution in the population became larger such that the heads of the Cannibal individuals became proportionately more tetragonal and the dorsal fins more shallow compared with the heads and fins of the NonCannibal individuals.

#### 3.4.2 Differentiation of morph types

**Figure 5:**
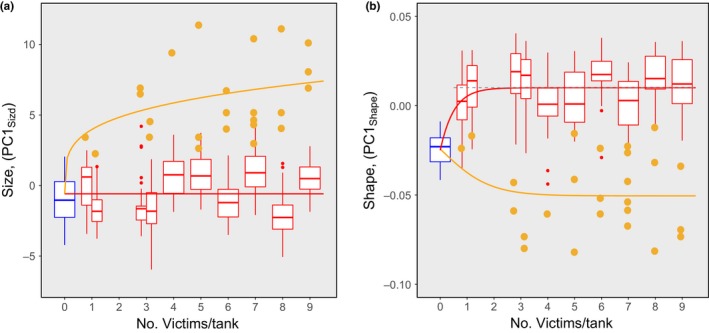
Size and shape differentiation in relation to the variation in the number of victims in each population. (a) Size differentiation. (b) Shape differentiation. The red box plots show the size and shape scores of NonCannibal individuals, and the blue box plots indicate the scores of Solitary individuals. The black dashed line in (b) shows the mean shape scores of NonCannibal individuals, based on the ANOVA results (Table 3). The solid red lines superimposed on the box plots represent the best description of the variation in size and shape of NonCannibal individuals in relation to the variation in the number of victims. The orange dots indicate size and shape scores of Cannibal individuals, and the orange solid lines superimposed on the orange dots are the best‐fit nonlinear curves based on the results nonlinear model selection analyses (see Table 4). See the text for more details

I considered the number of victims to be a proxy for the severity of cannibalism events in each population, and examined the size and shape differentiation of the populations with the change in the severity of cannibalism events. Figure 5 shows the size and shape differentiations in relation to the number of victims per tank. To describe how these differentiations advanced as the number of victims in the populations increased, I conducted *ad hoc* statistical look‐overs.

First, I focused on the size and shape of NonCannibals, and used analysis of variance (ANOVA) to compare the size (PC1_Size_ score) and shape (PC1_Shape_ score) variation among populations in relation to the number of victims. In the analyses, I treated Solitary individuals as hypothetical latent NonCannibal individuals in a zero‐victim population. PC1_Size_ scores did not differ between Solitary and NonCannibal (Table 3a), and I found neither a positive nor a negative linear regression effect related to the number of victims (Table 3a). These results imply that the sizes of Solitary and NonCannibal individuals neither increased nor decreased systematically with the number of victims (see Figure 5a). In contrast, PC1_Shape_ scores differed between Solitary and NonCannibal (Table 3b), although there was no significant variation among nonzero‐victim populations (Table 3b). These results imply that the shape changed drastically between Solitary and NonCannibal individuals, but that the mean shape of NonCannibals remained uniform regardless of the number of victims (see Figure 5b).

**Table 3:** Analysis of variance results for size scores, PC1_size_, and shape scores, PC1_Shape_, of NonCannibal individuals

Source of variation	SS	*df*	MS	*F*	*p <*
(a) Size					
Among groups	299.4894	10	29.9589	*F*_10,237_ = 10.5785	.0000
Solitary vs. NonCannibal†	7.6286	1	7.6286	*F*_1,237_ = 2.6936	.1021
Regression slope†	22.2588	1	22.2588	*F*_1,9_ = 0.7223	.4174
Deviation from regression	277.3306	9	30.8145	*F*_9,237_ = 22.238	.0000
Within groups	671.1996	237	2.8321		
(b) Shape					
Among groups	0.03828	10	0.003828	*F*_10,237_ = 15.9608	.000
Solitary vs. NonCannibal†	0.02912	1	0.02912	*F*_1,237_ = 121.4138	.000
Among nonzero victim groups†	0.00916	9	0.00102	*F*_9,237_ = 4.24371	.489
Within groups	0.05684	237	0.0002398		

Focal sources of variations in the analyses are shown by †. For more details, see the text.

Next, I focused on the size and shape of Cannibal individuals. I conducted separate best‐fit nonlinear model selection analyses of size and shape. In these analyses, I treated Solitary individuals as hypothetical latent Cannibal individuals in a zero‐victim population, and considered two competing nonlinear models, an asymptotic one and a nonasymptotic one. In the size analysis, the best model was a nonasymptotic increasing curve, and in the shape analysis, the best model was an asymptotic increasing curve (Table 4, see Figure 5).

**Table 4:** Nonlinear model selection analyses on size scores, PC1_Size_, and shape scores, PC1_Shape_, of Cannibal individuals

Model type	Function form of PC1_Size_	*k*	AIC	ΔAIC	*w*AIC
(a) Size					
Asymptotic	*a *+ *b*(1 − exp(−*c x*_2_))	3	244.7364	0.1403	0.4825
Nonasymptotic†	*a *+ *b x*_2_^*c*^	3	244.5961	0	0.5175

Model type	Function form of PC1_shape_	*k*	AIC	ΔAIC	*w*AIC
(b) Shape					
Asymptotic‡	*a *+ *b*(1 − exp(−*c x*_2_))	3	−285.8953	0	0.7180
Nonasymptotic	*a *+ *b x*_2_^c^	3	−284.0260	1.8693	0.2820

The explanatory variable, *x*_2_, is number of victims in each population. In each model, *k* parameters were estimated. The best models, shown by † and ‡, were evaluated using the weighted Akaike information criterion (*w*AIC). ^†^In the best model, the estimated parameters were *a *=* *−1.0682, *b *=* *4.5605, and *c *=* *0.2491. ^‡^In the best model, the estimated parameters were *a *=* *‐ 0.02455, *b *=* *−0.02580, and *c *=* *0.8223.

#### 3.4.3 Effect of interference and exploitation on the development of the cannibal morph

I expected the interference and exploitation events experienced to influence how and to what extent a given individual developed one or the other morph type, and I inferred that the Cannibal morph type is established in an individual when it dominates exploitation and interference experiences. Therefore, I focused on the largest Cannibal individual in each high‐density treatment tank and constructed six competing models to extract the inferred processes, that is, interference and exploitation experiences, using the numbers of Cannibal individuals (*x*_1_), and victims (*x*_2_) in each population to describe the size (PC1_Size_ score) and shape (PC1_Shape_ score) features of the largest Cannibal individuals.

The first three models were exploitation models, in which each variable *X*_*i*_, where *i* is the model number, indicates the number of victims exploited by the largest Cannibal individual in the population. Model 1 is an egalitarian exploitation model, in which variable *X*_1_ = *x*_2_ */x*_1_ is the number of victims exploited by the largest Cannibal individual if the victims were equally exploited by all Cannibal individuals in the population. Models 2 and 3 are exclusive exploitation models, in which variable *X*_*i*_ **= *x*_2_ − α_*i*_ *x*_1_ (where *i *=* *2 or 3) is the excess number of victims exploited by the largest Cannibal individual compared with the total number of victims exploited by other Cannibal individuals, each of which consumed α_*i*_ victims in the population. Model 2 is a semiexclusive exploitation model, in which α_2_ = 1 and *X*_2_ = *x*_2_ − α_2_ *x*_1_, and Model 3 is a completely exclusive exploitation model, in which the largest Cannibal individual exploits nearly all of the victims in the population; that is, α_3_ ≈ 0 and *X*_3_ **= *x*_2_ − α_3_ *x*_1_ (where α_3_ is set to 0).

The next three models (Models 4–6) were interference–exploitation models constructed by the systematic stepwise addition of *x*_1_, *x*_2_, and their interaction. I assumed that the number of Cannibal individuals (*x*_1_) was an indicator of the extent to which aggressive interference interactions occurred among Cannibal individuals in a population. Even though these models ignored the sequence in which events occurred and the time intervals between events, which would have influenced the states of the interacting population members, I still consider it worthwhile to examine them.

The size model that optimally described the PC1_Size_ scores of the largest Cannibal individuals was Model 3, the completely exclusive exploitation model (Table 5a). Of course, literal completely exclusive exploitation of all victims by the largest Cannibal individual is inconsistent with the existence of other Cannibal individuals in the tank. In fact, a cannibalism event involving each Cannibal individual in the population must have occurred at some time point in the past at which time the victim had reached some body size. Thus, the completely exclusive exploitation model should be interpreted here to indicate that the largest Cannibal individual had consumed most of the energy, whereas the other Cannibal individuals in the population had consumed smaller victims in the early part of the experimental period.

**Table 5:** Evaluation of the optimal models describing size scores, PC1_Size_, and shape scores, PC1_Shape_, of the largest Cannibal individuals in the populations of the high‐density treatment tanks as a function of several kinds of explanatory variables

Models	Function form of PC1_Size_	*k*	AIC	ΔAIC	*w*AIC
(a) Size					
1	*X*_1_ = *x*_2_/*x*_1_	2	54.0536	7.7810	0.0114
2	*X*_2_ = *x*_2_ – α_2_ *x*_1_	2	51.5745	5.3020	0.0395
3†	*X*_3_ = *x*_2_ – α_3_ *x*_1_	2	46.2726	0.0000	0.5598
4	*x*_1_	2	52.7792	6.5066	0.0216
5	*x*_1_ + *x*_2_	3	48.1799	1.9073	0.2157
6	*x*_1_ + *x*_2_ + *x*_1*_ *x*_2_	4	48.8814	2.6089	0.1519

Models	Function form of PC1_Shape_	*k*	AIC	ΔAIC	*w*AIC
(b) Shape					
1	*X*_1_ = *x*_2_/*x*_1_	2	−43.8363	8.4112	0.0122
2	*X*_2_ = *x*_2_ – α_2_ *x*_1_	2	−45.4578	6.7897	0.0275
4	*X*_3_ = *x*_2_ – α_3_ *x*_1_	2	−47.7712	4.4763	0.0875
3	*x*_1_	2	−44.8168	7.4307	0.0200
5	*x*_1_ + *x*_2_	3	−45.8042	6.4432	0.0327
6‡	*x*_1_ + *x*_2_ + *x*_1*_ *x*_2_	4	−52.2475	0.0000	0.8201

*x*_1_, number of Cannibal individuals, and *x*_2_, number of victims, in each population. Model 1, egalitarian exploitation model; Model 2, semiexclusive exploitation model (α_2_ = 1); Model 3, completely exclusive exploitation model (α_3_ = 0); Model 4, interference model; Model 5, interference + exploitation model; and Model 6, interference + exploitation + interference*exploitation model. In each model, *k* parameters were estimated. The best models, shown by † and ‡, were evaluated using the weighted Akaike information criterion (*w*AIC). ^†^The best model, PC1Size = 2.9872 + 0.855*X*_3_. ^‡^The best model, PC1_Shape_ = 0.02360 − 0.01593*x*_2_ − 0.03813*x*_1_ + 0.006031*x*_1*_ *x*_2_. For more details, see the text.

The shape model that optimally described the PC1_Shape_ scores of the largest Cannibal individuals was Model 6, which incorporates *x*_1_, *x*_2_, and their interaction (Table 5b).

**Figure 6:**
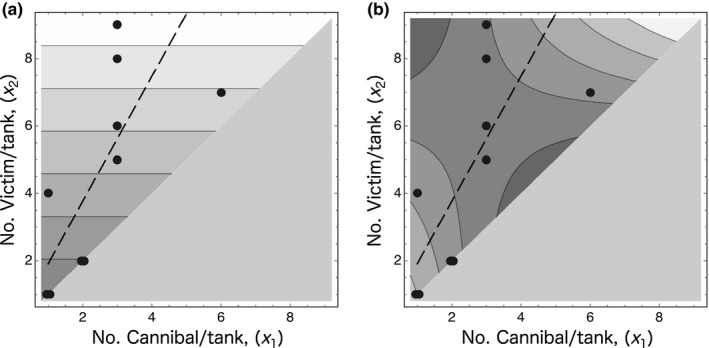
(a) Contour map of the optimal model fit to the size scores, PC1_size_, of the largest Cannibal individuals in the 10 experimental tanks of the high‐density treatment. Lighter areas indicate higher PC1_Size_ scores (larger size), and darker areas indicate lower PC1_Size_ scores (smaller size). (b) Contour map of the optimal model fit to the shape scores, PC1_shape_, of the largest Cannibal individuals in the 10 experimental tanks of the high‐density treatment. Lighter areas indicate higher PC1_Shape_ scores (less Cannibal‐type shape), and darker areas indicate lower PC1_Shape_ scores (more Cannibal‐type shape). The gray triangular areas occupying the lower right part of both maps are infeasible regions, given the assumption that no victims were divided and consumed by more than one Cannibal individual. The black dots on the maps show the census numbers of Cannibal individuals (*x*_1_) and victims (*x*_2_) in the experimental tanks, which have a correlative relationship, *r *=* *.7652. The dashed line on each map, indicates the linear relationship between *x*_1_ and *x*_2_ estimated by major axis regression of the observed (*x*_1_, *x*_2_) data (*x*_2_ = 0.05472 + 1.8559*x*_1_), describes the trend variation in *x*_1_ and *x*_2_

The selected model functions are shown by contours on the *x*_1_–*x*_2_ plane (number of Cannibal individuals vs. number of victims) in Figure 6. The change in the PC1_Size_ score along the trend variation line of *x*_1_ and *x*_2_ (dashed line in Figure 6a) shows that the size of the largest Cannibal individual increased with the number of victims in the population (*x*_2_), and most of these victims were assumed to have been consumed by the largest Cannibal individuals. The change in the PC1_Shape_ score along the trend variation line of *x*_1_ and *x*_2_ (dashed line in Figure 6b) shows that the largest Cannibal individuals had a more Cannibal‐type shape at intermediate points along the line.

## 4 DISCUSSION

### 4.1 Density‐driven expression of the cannibalism reaction norm

Expression of the cannibalistic reaction norm was driven by high density, which induced both a cannibalistic morph type and a noncannibalistic morph type, which was different from the low‐density morph type (Figure 4). The size–shape distributions of Cannibal and NonCannibal individuals in the high‐density treatment were subsumed into an identifiable highly correlated joint distribution, and each morph type was distributed at either end of the integrated distribution. In contrast, the size–shape distribution of Solitary individuals in the low‐density treatment was isolated from the joint distribution of Cannibal and NonCannibal individuals (Figure 3a). This result implies that in the development of the cannibalistic dimorphism, an individual's inducible morphology is subject to a common integration rule that is different from the rule inducing the Solitary morph type. Because the transition between the two developmental rules is a response to density, when expression of the cannibalism reaction norm is density‐driven, it can produce either a monomorphic population or a cannibalistic dimorphic population.

It can be reasoned that a reaction norm that can generate polymorphism among interacting population members is an evolutionary product, and that individuals of each morph type are adaptive in the population. Theoretical studies have demonstrated that adaptive state‐dependent induction of different phenotypes and life‐history variants produces an evolutionarily stable polymorphism (Gross, 1996; Maynard Smith, 1982). Expression of different phenotypes depending not only on an individual's own state but also on the states of other interacting individuals is ubiquitous in social situations (Frike & Frike, 1977; Godwin et al., 2003; Gross, 1991; Warner & Swearer, 1991; Wheerer, 1991). For example, in a situation involving conflict, expression of either an aggressive or a resistance phenotype depends not only on an individual's own state but also on the states of the other individuals.

Thus, the adaptive cannibalistic dimorphism‐generating reaction norm in *H. retardatus* larvae is such that individuals that grow rapidly to large size become aggressive cannibals and more slowly growing individuals become defensive. In fact, *H. retardatus* cannibal individuals have an aggressive nature (Nishihara, 1996a; Wakahara, 1995), and the NonCannibal individuals in the high‐density treatment showed a defensive response to the aggressive Cannibal individuals (unpublished data). Thus, density‐driven expression of the cannibalism reaction norm of this species is a state‐dependent adaptation that results in dimorphism. It would be possible to conclusively demonstrate that density‐driven expression of the cannibalism reaction norm controls the adaptive state‐dependent morphological differentiation to produce population dimorphism by tracking morphological developmental processes in individuals and morphological branching processes in the population of the high‐density experimental treatment.

### 4.2 Interaction‐driven expression of the cannibalism reaction norm

Even though a high population density induced dimorphism within a population, the state of the dimorphism differed among the populations. If within‐population contingent events are considered noise or unknowable, then these differences would likely be interpreted as random error in the populational expression of the reaction norm. From another perspective, the different populational states may be a result of heterogeneity of the provoked contingent social events among the populations.

In the cannibalism reaction norm, the number of actual cannibalism events within a population is a contextual clue to the effect of contingent social events on the induced populational dimorphism. As the number of victims within a population increased, the expression of the reaction norm changed such that the developed dimorphism became characterized by more highly correlative and skewed size–shape distributions (Figure 3b). Furthermore, when considered together with the “population” in which no contingent social events occurred (Solitary individuals), populational morphological distributions exhibited size and shape bifurcations along the gradient of cannibalistic contingent events (Figure 5).

Cannibals in a population are potential competitors in the exploitation of victims. In *H. retardatus*, one cannibal individual seemed to predominately consume conspecific victims, thus becoming the largest sized cannibal individual, in a sort of a contest‐type exploitative competition (Table 5a and Figure 6a). Furthermore, the shape of the largest cannibal, in particular, its enlarged, well‐developed jaw, reflects its function in this exploitative competition. However, the shape of the largest Cannibal individual reflected not only the consumption of victims in exploitative competition but also the effect of interactions with other Cannibal individuals in the population in interference competition (Table 5b). On the one hand, consumption of a large number of victims promoted induction of a more Cannibal‐type shape, but on the other hand, the simultaneous presence of many other Cannibal individuals in the population inhibited the induction of a more Cannibal‐type shape in the dominant individuals (Figure 6b). Thus, in a population with cannibal individuals, contest‐type exploitative competition occurs simultaneously with interference competition.

In the interaction‐driven expression of the cannibalism reaction norm, individuals may initially hesitate between an offensive or a defensive developmental pathway, depending on the occurrence of contingent social events, its own state, and the states of the other population members at every time point in the early developmental stage, before the individual is finally canalized to one or the other developmental pathway. These canalization processes are poorly understood; however, and to figure out their complexities among population members, it will be necessary to track social interaction events and phenotype development processes within a population.

Reaction norm evolution has been studied in the framework of fitness differences among individuals responding to the physical environment (Gomulkiewicz & Kirkpatrick, 1992; Schlichting & Pigliucci, 1995; Via & Lande, 1985). The social environment created by interactions among individuals also becomes a stage for the evolution of the reaction norm (Moore, Brodie, & Wolf, 1997). There may be social effects on phenotypic evolution whenever interacting phenotypes are present, and the fitness of an individual expressing a certain phenotype will be affected by the phenotypes of the individuals with which it is interacting. Several evolutionary genetics models (Kazancioglu, Klug, & Alonzo, 2012; McGlothlin, Moore, Wolf, & Brodie, 2010; Wolf, Brodie, & Moore, 1999) for the evolution of social phenotypes have been proposed, and some experimental studies have also demonstrated a genetic basis for interacting phenotypes and their evolution (Moore, Haynes, Preziosi, & Moore, 2002; Philippe et al., 2016).

In *H. retardatus*, the occurrence rate of cannibals in a high‐density environment varies among local populations (Michimae, 2006; Michimae & Wakahara, 2002), and local populations also show variation in other life‐history traits (Michimae, 2007; Michimae et al., 2009). These findings imply the existence of genetic variation among local populations. Phylogeographic studies have also identified genetic differences and phylogenetic relationships among local populations (Azuma, Hangui, Wakahara, & Michimae, 2013; Matsunami, Igawa, Michimae, Miura, & Nishimura, 2016; Michimae, 2006, 2007; Michimae et al., 2009).

Elaborate morphological analyses based on geometric morphometrics have shown that there exist shape differences in Cannibal, NonCannibal, and Solitary morph types among local populations (unpublished data). Different cannibalism reaction norms would result from a local population having evolved a different genetic basis for the reaction norm. The gene expression patterns in the predator‐ or prey‐induced morphological plasticity of *H. retardatus* larvae, which reflect the phenotypic expression mechanism, have been surveyed by a transcription analysis (Matsunami et al., 2015). In the future, I plan to explore the genetic basis of the cannibalism reaction norm in this species.

## CONFLICT OF INTEREST

None declared.

## AUTHOR CONTRIBUTIONS

KN did everything.
