# Supplementary figures and images for: An interaction‐driven cannibalistic reaction norm
Source: Ecol Evol. 2018 Jan 27;8(4):2305–19. doi: 10.1002/ece3.3801 (PMC5817123; doi:10.1002/ece3.3801)

Appendix Fig. A1

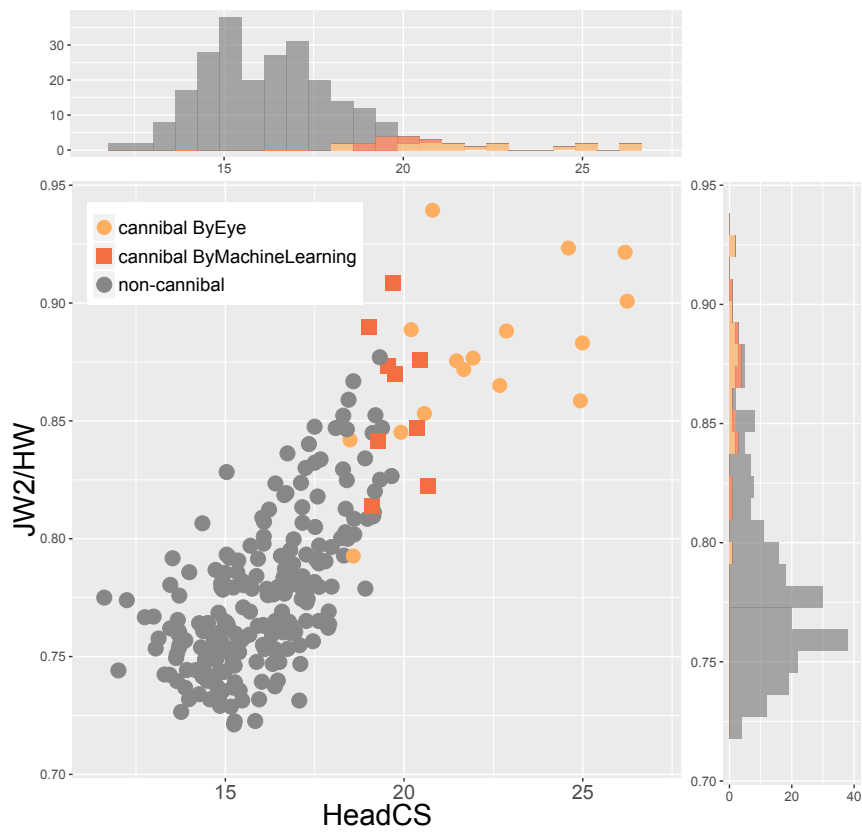

Supplement: Supplementary file 1 [file ECE3-8-2305-s001.pdf]
