# Supplementary material for: An interaction‐driven cannibalistic reaction norm
Source: Ecol Evol. 2018 Jan 27;8(4):2305–19. doi: 10.1002/ece3.3801 (PMC5817123; doi:10.1002/ece3.3801)
Supplement: Supplementary file 2 [file ECE3-8-2305-s002.docx]

Appendix Table A1. The classification model used by the semi-supervised machine-learning
classification algorithm for labeling the pre-labeled test data.

|  | Cannibal | NonCannibal |  |  | Cannibal | NonCannibal |
| --- | --- | --- | --- | --- | --- | --- |
| HW |  |  |  | HL2 |  |  |
| mean | 7.587 mm | 5.3897 mm |  | mean | 1.3604 | 1.1769 |
| sd | 0.8657 | 0.5297 |  | sd | 0.0635 | 0.0567 |
| JW1 |  |  |  | HL3 |  |  |
| mean | 5.2 | 3.0329 |  | mean | 4.9806 | 3.7172 |
| sd | 0.7971 | 0.4315 |  | sd | 0.3893 | 0.3783 |
| JW2 |  |  |  | SVL |  |  |
| mean | 6.701 | 4.1609 |  | mean | 18.0665 | 13.6412 |
| sd. | 0.8707 | 0.4936 |  | sd | 1.3662 | 1.1739 |
| EW |  |  |  | HeadCS |  |  |
| mean | 4.2418 | 2.8789 |  | mean | 22.4996 | 15.8324 |
| sd | 0.455 | 0.3089 |  | sd | 2.3151 | 1.539 |
| HL1 |  |  |  |  |  |  |
| mean | 1.272 | 0.9322 |  |  |  |  |
| sd | 0.1598 | 0.0977 |  |  |  |  |
