# Supplementary material for: An interaction‐driven cannibalistic reaction norm
Source: Ecol Evol. 2018 Jan 27;8(4):2305–19. doi: 10.1002/ece3.3801 (PMC5817123; doi:10.1002/ece3.3801)
Supplement: Supplementary file 3 [file ECE3-8-2305-s003.docx]

# **Appendix**: Supplemental screening for overlooked cannibals

## **Method**

In the initial visual judgment, I labeled a total of 16 individuals as “cannibals” with high credibility. However, on the basis of that judgment, no cannibals were identified in some tanks where cannibalism had in fact occurred. Therefore, I concluded that some cannibal individuals were missed by this first judgment.

I scanned the photographs of the individuals in each tank again, and selected an additional 24 ambiguous but likely cannibal individuals as test subjects, and then labeled the remaining 208 smaller individuals as “non-cannibals”. I then used a semi-supervised machine-learning classification algorithm (Chapelle *et al.*, 2006) to screen and detect cannibals among the test subjects.

As numeric input data, I used the lengths of certain body dimensions (HW, JW1, JW2, EW, HL1, HL2, HL3, and SVL; shown in Figure 1), and the head centroid size (HeadCS, see “Materials and Methods”). The supervised data consisted of the numeric attributes (the nine measured dimensions) of the 16 individuals initially labeled as “cannibals” and of the 208 individuals labeled as “non-cannibal” (labeled individuals), and the unsupervised test data consisted of the numeric attributes of the 24 individuals not yet labeled as either cannibal or non-cannibal (test data). First, an of-the-self classifier was trained by a naive Bayes method on the data of the labeled individuals. Second, the trained classifier was applied to the pre-labeled test data. The pre-labeled instances and the original labeled training data were then combined, and “cannibal” or “non-cannibal” was assigned to the pre-labeled instances by using the weighted k-nearest neighbor (k-NN) method (applying weight factor F = 1.0, and k = 10) with a KDTree (Pfahringer *et al.*, 2015). The classification algorithm was executed with the Weka ver. 3.7 software package (Bouckaert *et al.*, 2013).

## **Result**

The model used by the trained final classifier is shown in Table A1. Among the 24 test subjects, an additional nine individuals were labeled as “cannibals” and the remainder as “non-cannibals” by the classifier on the first screening. I conducted a provisional check of the performance of the final screening by examining the relationship between the JW2/HW ratio and the HeadCS (Figurer. A1). The positions of the newly identified cannibals relative to those of the previously labeled cannibals and non-cannibals suggests that the semi-supervised machine-learning classification functioned well.

# **References**

Bouckaert, R.R., Frank, E., Hall, M., Kirkby, R. & Reutemann, P. 2013. *WEKA Manual for Version 3-7-8*. The University of Waikato.

Chapelle, O., Schölkopf, B. & Zien, A. 2006. *Semi-supervised Learning*. MIT Press, Cambridge.

Nishihara, A. 1996. Effects of density on growth of head size in larvae of the salamander *Hynobius retardatus*. *Copeia* **1996**: 478.

Pfahringer, B., Driessens, K. & Reutemann, P. 2015. *Collective and Semi-supervised classification*. The University of Waikato.

Wakahara, M. 1995. Cannibalism and the resulting dimorphism in larvae of a salamander *Hynobius retardatus*, inhabited in Hokkaido, Japan. *Japanese Journal of Herpetology* **12**: 467–473.

Appendix Figure Legend

## Figure A1

Distribution of the classified cannibal and non-cannibal individuals with respect to the JW2/HW ratio and HeadCS. JW2/HW is a conventional shape index for discriminating between the “cannibal type” and the “non-cannibal type” (Wakahara, 1995; Nishihara, 1996). HeadCS is a simple size measure for discriminating cannibal-type individuals, which have an induced broad head morphology, from non-cannibal types. Light orange circles (cannibal ByEye) indicate individuals classified as cannibals on the basis of my initial visual judgment. Dark orange squares (cannibal ByMachineLearning) indicate individuals classified as cannibals by the semi-supervised machine-leaning classification algorithm. The gray circles indicate the individuals ultimately classified as non-cannibals.
